# Reform in Medical Education

**Published:** 1880-07

**Authors:** 


### EDITORIAL DEPARTMENT.

"If thou hast truth to utter, speak;
And leave the rest to God."

REFORM IN MEDICAL EDUCATION was the leading idea in our mind a few years ago, when we began the serious contemplation of a movement that culminated in obtaining an act of incorporation, and delivery of lectures in the last established medical school in this city. The original plan was, however, subordinated, it was hoped but temporarily, to expediency, as the necessity for obtaining lecturers who were willing to serve, even temporarily, was urgent, in order to begin the first session at the usual time of other schools. Thus the agitation of the pivotal motive of the enterprise, was rendered impracticable for some time afterward. The idea of reform then entertained, however, has not been materially changed by what has been effected in "the departure" of the leading schools of this country, in their increased number of sessions, and graded courses. Our purpose was to organize a school with not *less than six nor more than eight* professors, who should be required to lecture or otherwise teach their respective branches for a period of one hour four times a week, during a continuous session or course of *nine* months in each year; and that it should be obligatory upon candidates for the doctorate to establish the fact of regular attendance upon *all* the branches taught during two of these sessions at least. It was thought that this arrangement would give ample time for lectures on special subjects, clinical and otherwise, dissections, etc., but

"The best laid schemes *o'mice* an' *men*,

Gang aft a-gley,

An' lea'e us nought but grief and pain,

For promis'd joy."

Failing to secure the co-operation of several prominent gentlemen to whom appeal was made to join us, with two or three others, as corporators, most of whom it was expected would become teachers also, in the school, we were *compelled to take whom we could get*, in order to the carrying out the then usual programme of lectures during the rapidly approaching session. But unfortunately for the consummation of *our* "project of reform," we were soon made to realize that *those* parties had *votes* as well as ourself, and which were as potent in any contest that might arise, as were the votes of others who might desire the success of the school upon the original idea, that gave it birth.

Finding that it would be impossible to harmonize with the majority, even in carrying out the *original* compact concerning the admission of new members into the faculty, a gentleman whom we had asked to unite with us in the new enterprise, ere *any other* person was requested to do so, resolved like ourself, to retire from the concern, and our resignations were accordingly announced. But we must conclude our remarks for the present, particularly, as we have given a quasi promise to write a history of the school from its inception to the present time. As it has ceased to be a *private* affair, and *belongs* now to the *public* of Maryland, we expect, should time permit us to write as requested, to speak of it as such; and therefore, through another medium than a medical journal, as the laity will *probably* be as *much interested* in its history as the profession.

